# The Individualized Genetic Barrier Predicts Treatment Response in a Large Cohort of HIV-1 Infected Patients

**DOI:** 10.1371/journal.pcbi.1003203

**Published:** 2013-08-29

**Authors:** Niko Beerenwinkel, Hesam Montazeri, Heike Schuhmacher, Patrick Knupfer, Viktor von Wyl, Hansjakob Furrer, Manuel Battegay, Bernard Hirschel, Matthias Cavassini, Pietro Vernazza, Enos Bernasconi, Sabine Yerly, Jürg Böni, Thomas Klimkait, Cristina Cellerai, Huldrych F. Günthard, The Swiss HIV Cohort Study

**Affiliations:** 1 Department of Biosystems Science and Engineering, ETH Zurich, Basel, Switzerland; 2 SIB Swiss Institute of Bioinformatics, Basel, Switzerland; 3 Division of Infectious Diseases and Hospital Epidemiology, University Hospital Zurich, University of Zurich, Zurich, Switzerland; 4 Clinic for Infectious Diseases, Bern University Hospital, Bern, Switzerland; 5 Division of Infectious Diseases and Hospital Epidemiology, University Hospital Basel, Basel, Switzerland; 6 Division of Infectious Diseases, Geneva University Hospital, Geneva, Switzerland; 7 Division of Infectious Diseases, University Hospital Lausanne, Lausanne, Switzerland; 8 Division of Infectious Diseases, Cantonal Hospital St. Gallen, St. Gallen, Switzerland; 9 Division of Infectious Diseases, Regional Hospital Lugano, Lugano, Switzerland; 10 Laboratory of Virology, University Hospital Geneva, Geneva, Switzerland; 11 Swiss National Center for Retroviruses, Institute of Medical Virology, University of Zurich, Zurich, Switzerland; 12 Institute for Medical Microbiology, University of Basel, Basel, Switzerland; 13 Division of Immunology and Allergy, Centre Hospitalier Universitaire Vadois, Lausanne, Switzerland; Katholieke Universiteit Leuven, Belgium

## Abstract

The success of combination antiretroviral therapy is limited by the evolutionary escape dynamics of HIV-1. We used Isotonic Conjunctive Bayesian Networks (I-CBNs), a class of probabilistic graphical models, to describe this process. We employed partial order constraints among viral resistance mutations, which give rise to a limited set of mutational pathways, and we modeled phenotypic drug resistance as monotonically increasing along any escape pathway. Using this model, the individualized genetic barrier (IGB) to each drug is derived as the probability of the virus not acquiring additional mutations that confer resistance. Drug-specific IGBs were combined to obtain the IGB to an entire regimen, which quantifies the virus' genetic potential for developing drug resistance under combination therapy. The IGB was tested as a predictor of therapeutic outcome using between 2,185 and 2,631 treatment change episodes of subtype B infected patients from the Swiss HIV Cohort Study Database, a large observational cohort. Using logistic regression, significant univariate predictors included most of the 18 drugs and single-drug IGBs, the IGB to the entire regimen, the expert rules-based genotypic susceptibility score (GSS), several individual mutations, and the peak viral load before treatment change. In the multivariate analysis, the only genotype-derived variables that remained significantly associated with virological success were GSS and, with 10-fold stronger association, IGB to regimen. When predicting suppression of viral load below 400 cps/ml, IGB outperformed GSS and also improved GSS-containing predictors significantly, but the difference was not significant for suppression below 50 cps/ml. Thus, the IGB to regimen is a novel data-derived predictor of treatment outcome that has potential to improve the interpretation of genotypic drug resistance tests.

## Author Summary

Drug resistance remains a challenge in the management of HIV-infected patients. The accumulation of mutations during ongoing viral replication is the origin of drug resistance development. Understanding this evolutionary process in a quantitative manner is an important prerequisite for minimizing the risk of resistance development and for the optimal selection of drug combinations for each individual patient. We present probabilistic graphical models for describing the evolution of drug resistance, and we derive the individualized genetic barrier (IGB), a single quantity summarizing the genetic potential of the virus for evolutionary escape from selective drug pressure. The predictive power of the IGB is demonstrated on a large well characterized clinical cohort of HIV patients and compared to classical predictors.

## Introduction

Despite an increasing arsenal and improved potency of antiretroviral drugs, the optimal use of combination antiretroviral therapy against HIV-1 infection remains challenging [1]. Complicating factors include drug interactions and toxicities, adherence to therapy, and development of drug resistance [2]. Because genotypic drug resistance testing is performed on a routine basis today and because mutational patterns are unique for each patient, treatment choices are, in principle, highly personalized. In practice, however, it can be difficult to identify an optimal drug combination for each individual patient due to the combinatorial complexity of both the set of feasible drug combinations and of viral mutational patterns.

In addition to controlled clinical trials, analyzing data from large observational cohort studies is a promising way to identify predictors of treatment outcome, even if the availability of drugs and therapeutic strategies change over time [3]. This approach can be based on modeling the risk of acquiring additional mutations [4], on estimating future drug options [5], on predicting the time to virological failure [6], [7], or on classifying the regimens of treatment change episodes (TCEs) as successful versus failing, depending on the patient's response to therapy. A TCE consists of predictor variables including the applied drug combination, viral genotype, treatment history, demographic and clinical parameters, and a response variable such as the change in viral load.

HIV-1 genotype has been shown to be a strong predictor of therapeutic success in retrospective and prospective studies [8]–[14], but the large number of mutations complicates prediction. TCE classification is a noisy, high-dimensional prediction problem with unobserved confounding factors and sparse data. It has been addressed by several statistical learning methods [15]–[25]. Comparative studies have emphasized the importance of selection and representation of features, especially of the viral genotype, over the choice of the learning algorithm [26]–[28]. In order to directly correlate genotype with clinical response, rules-based approaches, such as the genotypic susceptibility score (GSS) [29]–[34] and statistical models [23], [26], [28] have been proposed, often outcompeting human experts [35].

Drug resistance development is driven by viral evolution and thus models of viral evolutionary escape from drug pressure have been proposed to improve therapy response prediction [16], [22], [36]. Specifically, the individualized genetic barrier (IGB) to drug resistance has been suggested as a predictor of treatment outcome. The IGB is defined as the probability of the virus not to become resistant to a certain drug [37]–[39]. A high IGB means that viral evolutionary escape from the selective pressure of the drug is unlikely. Related quantities are the average number of mutations and the average time to reach drug resistance derived from simulated HIV-1 evolutionary trajectories on an estimated fitness landscape [36], [40], [41]. This approach has been explored for treatment with zidovudine plus lamivudine and with nelfinavir [42], but it does not scale to the variety of combination therapies observed in clinical databases, because sufficient data for estimating fitness landscapes is available only for a few drug combinations. Earlier, the term ‘calculated genetic barrier’ has been used to assess the number of mutations necessary to acquire specific drug resistance-associated mutations, which were found to be similar among HIV-1 subtypes [43].

In the present study, we apply a simplified definition of the IGB which can be computed efficiently for any drug combination based on a statistical model that captures the order and the dynamics of accumulating mutations and the associated levels of phenotypic drug resistance [44]. The IGB to resistance to a certain drug is the probability that the virus will not accumulate additional mutations leading to a resistant strain. This drug-specific IGB has been demonstrated to be a strong predictor of virological response in two large observational cohort studies [26], [28]. Here, we derive a novel predictor, the IGB to the entire drug combination which measures the genetic potential for evolutionary escape of the virus from the selective pressure of combination therapy.

In order to assess the performance of the IGB as a predictor of treatment outcome, we analyzed TCE data from the Swiss HIV Cohort Study (SHCS) database, a large, long-term observational, multi-center, clinical database with integrated results of genotypic drug resistance tests [45], [46]. We identified risk factors of therapeutic failure and constructed models of treatment outcome considering as predictors the applied regimen, treatment history, viral genotype, GSS, drug-specific IGBs, IGB to regimen, and demographic and clinical variables including patient adherence. Overall, we found the IGB to the entire regimen to be the strongest and most significant predictor. Our results demonstrate that the viral genotype is represented efficiently by the IGB to regimen, a single, interpretable probability summarizing the predicted dynamics of viral evolutionary escape.

## Results

**Figure 1:**
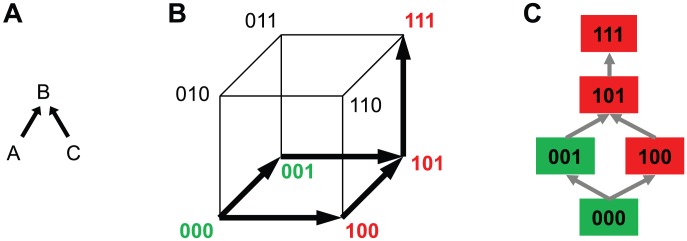
Schematic illustration of I-CBN model and individualized genetic barrier (IGB). (A) A partially ordered set of three mutations, 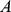, 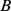, and 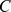, is considered with the two relations 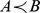 and 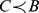, resulting in two possible escape pathways of the virus, namely 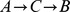 or 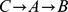. (B) The partial order constraints give rise to the genotype lattice consisting of genotypes 000, 001, 100, 101, and 111 indicated with bold arrows, where genotypes are encoded as binary strings such that 000 is the wild type 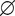 (no mutations), 100 is defined by mutation 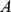 and identified with 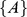, 101 with 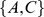, etc. The genotype lattice 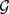 is shown inside the embedding hypercube 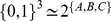. For each antiretroviral drug, genotypes are labeled as either susceptible (green) or resistant (red). (C) Genotype lattice isolated from the embedding hypercube. The IGB is the probability of the virus not reaching a resistant state.

For each drug, viral evolutionary escape from its selective pressure was modeled using Isotonic Conjuctive Bayesian Networks (I-CBNs). In these probabilistic graphical models, dependencies among mutations are described by a partial order, which defines the genotype lattice, i.e., the set of genotypes compatible with the order constraints, and hence the set of possible mutational escape pathways (Figure 1). To each genotype, its level of phenotypic drug resistance is associated using isotonic regression, such that drug resistance is monotonically non-decreasing along any mutational pathway from the wild type towards the genotype carrying all mutations. Using cross-sectional matched genotype-phenotype pairs from the Stanford HIV Drug Resistance Database, I-CBN models were learned for a total of 18 antiretroviral drugs (Supporting Figures S4, S5, S6, S7, S8, S9, S10, S11, S12, S13, S14, S15, S16, S17, S18, S19, S20, S21, Supporting Table S2). Each model includes up to eleven pre-selected mutations (see Methods).

**Figure 2:**
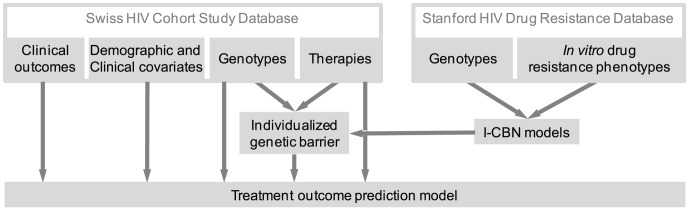
Data flow. Matched pairs of viral genotype and drug resistance phenotype from the Stanford HIV Drug Resistance Database (top right) were used to learn I-CBN models for all drugs separately. The drug-specific individualized genetic barriers (IGBs) are derived from these models. The IGB to regimen is computed for each genotype-therapy pair in the Swiss HIV Cohort Database and its predictive power is assessed in prediction models that also account for classical demographic, clinical, and genetic covariates.

From the I-CBN models, transition probabilities among genotypes were derived and the individualized genetic barrier (IGB) to resistance development to each drug was computed as the probability of the observed genotype not acquiring additional mutations that would transform it into a genotypic state predicted to be resistant. For a drug combination, the IGB was obtained as the sum over all drugs of the regimen of the drug-specific IGBs. Thus, the IGB to regimen can be regarded as the expected number of active components in the drug cocktail taking viral evolutionary escape mechanisms into account. To assess the predictive power of the IGB in a clinical setting, we analyzed a large cohort of HIV-1-infected patients and compared the IGB to several known predictors of therapy response (Figure 2), including the GSS, obtained from the Stanford HIV Drug Resistance Database website (HIVdb 6.2.0).

TCEs from the time period 1988–2010 were derived from the SHCS database (Table 1 and 2) and labeled as either failure or success (see Methods). Therapy success was defined as viral load reduction below 50 cps/ml (400 cps/ml) during treatment. We obtained 2185 (2631) genotype-therapy pairs, including 73% (63%) failures. The usage of individual drugs and the 30 most frequent drug combinations are shown in Supporting Figures S2S and S3, respectively. The historical development of drug usage patterns is reported in Supporting Table S3, where the regimens are annotated as either being recommended as first-line or alternative regimens according to current treatment guidelines [47], or as past first-line or second-line recommended regimens that are still in use in developing countries or occasionally used if drug resistant virus is present at baseline or as salvage regimens, or as regimens that are not in use anymore as first-line regimens but were before, including those still used under special circumstances, such as unusual tolerability.

**Table 1:** Characteristics of the numerical predictors in the SHCS database.

	50 cps/ml		400 cps/ml	
Numerical variables	median	(IQR)	median	(IQR)
Age	40	(35–46)	40	(35–46)
Minimum CD4 T cell count (cells/ 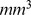 )	108	(40–200)	110	(40–206)
Maximum viral load (log10 copies/ml)	5.17	(4.72–5.63)	5.15	(4.66–5.61)

**Table 2:** Characteristics of the categorical predictors in the SHCS database.

		50 cps/ml		400 cps/ml	
Categorical variables		frequency	(%)	frequency	(%)
Gender	female	562	(25.72%)	705	(26.8%)
	male	1623	(74.28%)	1926	(73.2%)
AIDS	no	1461	(66.86%)	1775	(67.46%)
	yes	724	(33.14%)	856	(32.54%)
Transmission group	blood	16	(0.73%)	27	(1.03%)
	heterosexual	719	(32.91%)	881	(33.49%)
	IDU	491	(22.47%)	598	(22.73%)
	male homosexual	879	(40.23%)	1033	(39.26%)
	mother-to-child	12	(0.55%)	16	(0.61%)
	others/unknown	68	(3.12%)	76	(2.88%)
Ethnic Group		9	(0.41%)	10	(0.38%)
	asian	41	(1.88%)	58	(2.2%)
	black	281	(12.86%)	347	(13.19%)
	hispano american	34	(1.56%)	46	(1.75%)
	white	1743	(79.77%)	2080	(79.06%)
	unknown	77	(3.52%)	90	(3.42%)
Adherence to treatment	low	496	(22.7%)	610	(23.19%)
	high	1586	(72.59%)	1899	(72.18%)
	others/unknown	103	(4.71%)	122	(4.64%)

In order to predict the outcome (failure versus success) of each therapy, we considered applied drugs, demographic and clinical variables, viral genotype, IGBs to received drugs, and IGB to regimen (Figure 2, Table S1). Univariate logistic regression resulted in a total of 50 (44) features that were significantly associated with therapy outcome (Figure S22). Among the predictive drugs, the use of ZDV, d4T, 3TC, and NFV were associated with increased risk of therapeutic failure, while ABC, TDF, FTC, EFV, RTV, LPV/r, ATV, and ATV/r increased the odds of therapeutic success. Most of the significant amino acid changes in the viral protease (PR) gene (10I, 30N, 33F, 46I, 54V, 71V, 82A, 84V, 90M) and reverse transcriptase (RT) gene (39A, 41L, 44D, 67N, 74V, 103N, 118I, 123S, 210W, 215Y, 297R) have been associated with resistance to multiple PR inhibitors (PIs) and RT inhibitors (RTIs), respectively, and all except PR 30N and RT 123S increased the risk of treatment failure. A higher IGB to any of 15 (16) individual drugs increased the chance of successful virological response. The IGB to the entire drug combination and the GSS were also significant predictors.

**Figure 3:**
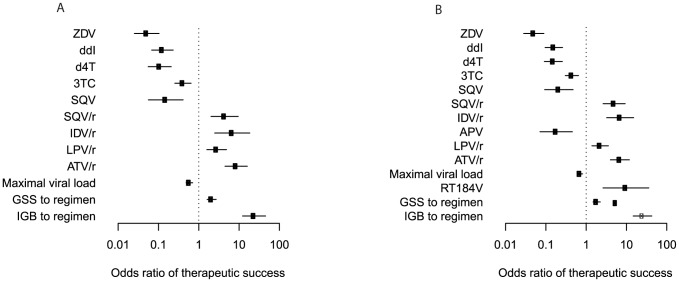
Multivariate analysis of predictors of response to antiretroviral combination therapy in the SHCS database. Associations have been tested using a logistic regression model and odds ratios of therapeutic success, defined as viral load reduction below 50 cps/ml (A) and 400 cps/ml (B), are reported together with their 95% confidence intervals on a logarithmic scale. Benjamini-Hochberg-corrected p-values are represented as black (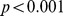) and grey (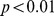) symbols. Only predictors with a p-value smaller than 0.01 are included.

In the multivariate analysis, only 12 (14) variables were significant, nine (ten) of which are indicating the inclusion of individual drugs in the regimen (Figure 3). The usage of the nucleoside RTIs (NRTIs) ZDV, ddI, d4T, and 3TC, and of the PIs APV and SQV, were associated with negative treatment outcome, whereas the four boosted PIs (i.e., given together with low-dose RTV to improve their bioavailability) SQV/r, IDV/r, LPV/r, and ATV/r had positive predictive power. Among the many genotype-derived predictors, only GSS and IGB to regimen reached statistical significance at the 1% level in the multivariate model. For the 50 cps/ml success definition, the odds ratio (OR) of therapeutic success was ten-fold higher for the IGB (OR 23.6, 95% confidence interval [CI] 12.21–45.4, 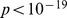) as compared to the GSS (OR 2.1, 95% CI 1.6–2.7, 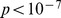), and similarly for 400 cps/ml (IGB OR 25.0, 95% CI 14.7–42.5, 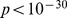 versus GSS OR 1.8, 95% CI 1.5–2.2, 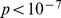), indicating that the IGB provides an effective summary of the risk of treatment failure due to viral genetic changes. In addition, increased overall maximum (peak) viral load before treatment remained a significant predictor of therapy outcome in the multivariate logistic regression model.

**Figure 4:**
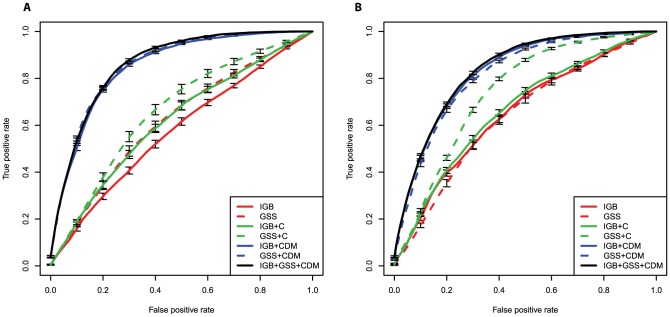
ROC curves quantifying the performance of elastic net regularized logistic regression models in predicting treatment outcome, defined as a reduction of viral load below 50 cps/ml (A) and 400 cps/ml (B). The areas under the ROC curves (AUC values) are reported in Table S5 and Table S6. Prediction models are encoded by the sets of predictors used, where C refers to the demographic and clinical variables, D refers to drugs, and M to mutations. For example, the model IGB+CDM includes as predictors IGB to regimen, clinical and demographic predictors, applied drugs, and mutations. The models with all predictors perform significantly better than all other models.

For optimal treatment outcome prediction, we also explored the use of regularized logistic regression models. Specifically, the elastic net, which combines 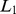 and 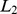 regularization was applied to identify sparse classifiers of therapy outcome. Classifier performance was evaluated in ROC curves summarized by the area under the ROC curve (AUC), and analyzed according to the historical drug usage patterns (Table S3).The competitive models (high AUC) are only those using all clinical and demographic variables, mutations, and drugs (Tables S5, S6, Figure 4). When comparing IGB to GSS as predictors in this setting, we found a significant advantage of the IGB for 400 cps/ml if all other features are included in the models (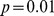, Wilcoxon rank sum test). Furthermore, the IGB also improves treatment outcome prediction if added to models that already contain the GSS (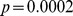). For 50 cps/ml, we did not find significant differences in AUC between IGB and GSS when used in prediction models that included all other covariates, nor did the GSS-containing model improve upon adding IGB. The significant increase for the larger dataset with the 400 cps/ml success definition demonstrates the predictive power of the IGB and indicates that GSS and IGB, although correlated, contain some orthogonal information, which, if combined, can further improve treatment outcome prediction.

## Discussion

We have comprehensively analyzed factors of therapy outcome in the SHCS database using univariate, multivariate, and regularized multivariate logistic regression models. As predictors of therapeutic success we identified the applied drugs, the GSS, and as the strongest predictor the IGB to regimen, a novel predictor derived from viral genotype.

Including genotype information into treatment outcome prediction is challenging because of the large number of observed mutations and the complexity of the genotype-phenotype relationship. Here, we have explored the IGB to drug resistance as a summary measure of the escape dynamics of the virus under treatment. The underlying idea of this modeling approach is that the IGB captures how difficult it is for the virus to escape from the selective pressure of individual drugs or from the entire drug combination. This piece of information is different from assessing the current genotypic or phenotypic drug resistance state of the virus, as intended, for example, by the GSS. The IGB makes a prediction about the expected escape dynamics of the virus population given its current genetic state.

The computation of the IGB involves an evolutionary model of genetic progression under selective drug pressure along multiple mutational pathways and a notion of evolutionary escape, which was based here on the predicted level of phenotypic drug resistance. We applied I-CBN models for jointly describing genetic progression and associated phenotypic change of the virus. In particular, phenotype predictions are non-linear in the mutations, which allows for capturing epistatic effects, i.e., the same mutation can have different effects on the resistance phenotype depending on the genetic background of the virus (Figure 1). The I-CBN models were estimated from independent genotype-phenotype data. Using these models, the complex, high-dimensional, genotypic data of each virus can be summarized efficiently by the IGB to resistance to each drug. Thus, rather than modeling interactions between drugs and individual mutations, the IGB provides a comprehensive model of drug-genotype interaction.

In the present study, we have extended the concept of the IGB to the entire regimen in a fashion that allows for computing this quantity for any drug combination and hence for large clinical datasets. The IGB to regimen can be regarded as the expected number of active drugs in the regimen. Assuming independent effects among drugs, we compute the regimen IGB from the drug IGBs. These simplifying assumptions are made for computational feasibility. They present a conceptual limitation of the approach and more elaborate models are conceivable. In addition, other variables not included in this study might be important, for example, pharmacological properties of drug combinations and host genetic factors. Here, the IGB, a single interpretable quantity, was found to be the strongest genotype-derived predictor of virological response and hence the most efficient representation of the viral genotype with respect to therapy outcome.

We have used throughout two definitions of virological success of treatment, namely reduction of viral load below 50 cps/ml and below 400 cps/ml. The latter less stringent cutoff was included because in the past it represented the limit of detection of viral load assays. Today viral load values of 50 cps/ml and lower can be measured and reduction below 50 cps/ml (or below the limit of detection) is an accepted therapeutic goal. We generally found very similar results for the two datasets, but the advantage of using IGB over GSS (the de facto standard genotype interpretation tool) reached statistical significance only for 400 cps/ml, but not for 50 cps/ml. This finding may, in part, be due to the larger dataset and hence increased statistical power for 400 cps/ml as compared to 50 cps/ml. In the future, larger datasets will be required to further evaluate the IGB and its potential to predict treatment outcome without the need for expert rules. This property of the IGB is particularly appealing for new drugs, for which reliable rules are not readily available before evidence has accumulated in published studies. Larger datasets and more elaborate statistical variable importance methods [48] will also increase the power to detect other factors of therapeutic outcome, but the general consistency between the 50 cps/ml and 400 cps/ml success definitions suggests that a sizable fraction of important variables have been identified. In addition, larger TCE databases will allow for analyzing alternative endpoints, such as time to virological failure or virological response after a fixed period of time.

In the univariate analysis, most drugs had a positive effect on treatment outcome, with the exception of ZDV, d4T, 3TC, and NFV. The negative associations might be due to the prominent use of the drug combinations (ZDV or d4T) +3TC+ (IDV or NFV), 90% of which were failures. The four drugs were among the first to be approved for antiretroviral therapy and used in early suboptimal regimens. Moreover, they were poorly tolerated and therefore one can expect a general lower adherence to treatment. A similar observation was made in the multivariate analysis, where ZDV, ddI, SQV, 3TC and d4T were significant predictors decreasing the odds of therapeutic success. This effect might also be due to the common early use of these drugs in mono therapy and their later use in salvage regimens, even if multiple resistance mutations had already accumulated [49]. Among PIs, a pronounced trend was that boosting with RTV increased the odds of successful treatment. The fraction of PI boosting in the dataset is reported in Supporting Table S4.

A few variables did not show significant association with therapy outcome although they might have been expected to. For example, adherence is a well-known predictor of treatment success [50], [51], but it failed to reach significance in the multivariate model, most likely due to lack of adherence data for about 45% of the patients. The missing data resulted from collecting adherence data within the SHCS only since January 2003. Indeed, in a multivariate analysis restricted to the subset of 1183 TCEs with observed adherence a more pronounced effect can be observed. We have not included a set of variables in this study that are known to be predictors because of the construction of the dataset. The definition of the dataset of genotype-therapy pairs allows for including several sequential TCEs from the same patient. Most TCEs are actually derived from unique patients, but some patients occur multiple times. Each TCE gives rise to two therapy cases, a failure, which had given rise to the switch, followed by a salvage regimen, which can be a failure or a success. Therefore, we did not include variables that are affected by the sequential ordering of therapies, such as the total time a patient was under therapy with a certain drug or the calendar year of treatment.

In summary, the IGB to regimen is a new predictor of treatment outcome that captures, in a single quantity, the virus' genetic potential for developing drug resistance under the selective pressure of the combination therapy. The IGB can be computed efficiently for any viral genotype and any drug combination. It may thus contribute to improved interpretation of genotypic drug resistance tests and to the rational design of individualized therapies. Future prospective studies are required to apply these results to other patient populations and to eventually integrate them into clinical practice.

## Methods

### Swiss HIV Cohort Study (SHCS) database

Founded in 1988, the SHCS is a nationwide, prospective, multicenter, clinic-based cohort with continuous enrolment and semi-annual study visits representing approximately 50% of all HIV-infected and 75% of all treated patients in Switzerland [46]. The SHCS has been approved by ethical committees of all participating institutions, and written informed consent has been obtained from all participants. The SHCS drug resistance database contains the results of 13,201 genotypic resistance tests from 9,231 patients, stored in a central database [45]. Resistance data stem from routine clinical testing (60%) and from tests performed retrospectively from frozen repository plasma samples (40%) (Table 1 and 2).

The SHCS has been approved by the following ethical committees of all participating institutions: Kantonale Ethikkommission Bern; Ethikkommission beider Basel; comité d'éthique du département de médicine de Hôpitaux Universitaires de Genéve; commission d'éthique de la recherche clinique, Lausanne; comitato etico cantonale, Bellinzona; Ethikkommission des Kanton St.Gallens; and Ethik-Kommission Zürich, all Switzerland. Written informed consent has been obtained from all participants [46].

### Treatment change Episode (TCE) data

TCEs were obtained from the SHCS database as follows. Each TCE consists of a failing therapy followed by a salvage therapy (Supporting Figure S1). We required that the failing therapy was at least four month long and that the genotype was measured no more than 90 days before and no more than 30 days after onset of the uninterrupted salvage therapy [26]. In order to restrict to failing regimens due to viral rebound and to exclude convenience treatment changes or single determinations of low-level viremias (blips), a failing therapy was defined by either two consecutive viral load measurements above 500 cps/ml, or a single viral rebound followed by therapy switch, or single rebound after 180 days and lack of viral suppression below the limit of detection.

Therapies were labeled ‘success’ versus ‘failure’ as follows. Any failing therapy was considered a failure. Salvage therapies were considered successful, if viral load dropped below 50 cps/ml at any time point during treatment, otherwise they were considered failures. Because viral load assays with a sensitivity of 50 cps/ml were not available for the whole observation period, we also considered an alternative definition of therapy success as a viral load reduction below 400 cps/ml. The TCE dataset spans the time period 1988–2010, but 75% of TCEs date from 2000 or later.

### Isotonic Conjunctive Bayesian Network (I-CBN) models

Genetic progression of the virus under selective drug pressure and the resulting phenotypic drug resistance changes were modeled jointly using I-CBNs [44]. In this model, mutations occur subject to partial order constraints which define the genotype lattice, the set of genotypes compatible with the constraints, and drug resistance is non-decreasing along any mutational pathway (Figure 1). Formally (see [44] for details), let 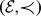 be a partially ordered set of 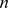 mutations. Each genotype is identified with the subset 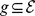 of mutations it carries. The genotype lattice 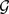 induced by 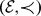 is the set of all genotypes 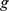 for which it holds that 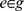 implies 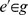 whenever 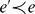 in 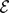. We denote by 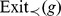 the set of accessible mutations from genotype 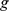 under the given partial order constraints. The I-CBN is a statistical model for the random variables 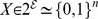, describing observed genotypes, and 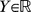, describing associated drug resistance phenotypes, both of which are observed from true hidden genotypes 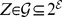 subject to noise. The probability of an unobserved genotype 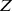 is defined as
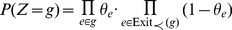
(1)where the parameters 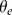 denote the conditional probabilities of mutation 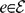 given that all of its predecessor mutations have occurred, 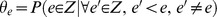. The observed random variables 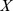 and 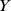 are independent given 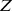. The genotype observation error is modeled as
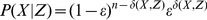
(2)where 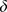 denotes the Hamming distance and errors are assumed to occur independently among sites at rate 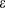. The observed drug resistance phenotype 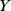 is the log fold-change in susceptibility. For each genotype 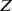, it follows a normal distribution
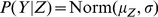
(3)subject to the monotonicity contraints 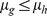 for all genotypes 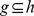. The complete model for 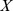 and 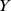 is then the marginalization
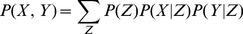
(4)Parameter estimation for this model was performed using the EM algorithm described in [44].

The model was applied separately to 18 antiretroviral drugs, using between 280 and 2303 (median 1448) cross-sectional genotype-phenotype pairs, i.e., observations of 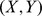, obtained from the Stanford HIV Drug Resistance Database, restricted to subtype B sequences and to Phenosense or Antivirogram assays [52]. For each drug, we selected its resistance-associated mutations reported on the Stanford HIVdb website lumping together mutations occurring at the same site, or if unavailable, applied 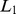-penalized (lasso) linear regression [53], [54] to select from all PR or RT mutations occurring at least ten times a sparse set 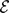 of 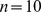 predictor mutations. The performance of the models is reported as the Pearson correlation coefficient between true and predicted phenotypes, estimated from a separate, random subset of 20% of the data. Phenotypic cutoff values were derived from the distribution of fold-change values as described previously [15], [26] and used to dichotomize resistance predictions (Supporting Table S2).

### Individualized Genetic Barrier (IGB)

Given an I-CBN model, transition probabilities among genotypes 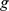, 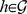 can be computed as
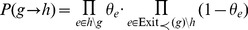
(5)Using these transition probabilities and the predicted drug resistance phenotypes 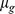, we define the IGB of genotype 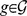 to resistance to drug 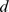 as the probability of the virus not reaching any genotypic state predicted as resistant,
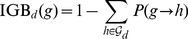
(6)where 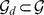 is the subset of all genotypes 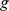 predicted to be resistant to drug 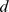, i.e., for which 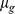 is greater than the resistance cutoff (Supporting Table S2).

Genotypes outside the lattice 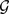 (not complying with the partial order constraints) are regarded as erroneous observations of the genotypes in the lattice. The IGB of such a genotype 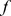 is
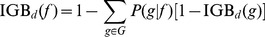
(7)where 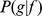 is the probability of the actual genotype being 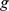 given that 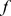 has been observed. By Bayes' theorem,
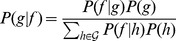
(8)where 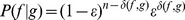 is modeled as in Eq. 2.

The genetic barrier to escape from a regimen 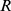 is defined as the sum of the drug-specific barriers over all drugs in the regimen
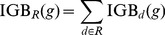
(9)Because the IGB to each drug can be regarded as an estimate of the activity of the drug (the probability of not escaping), the IGB to a regimen may be interpreted as the expected number of active drugs in the regimen. Note that 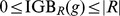, that 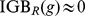 means that evolutionary escape is almost certain, and that adding a drug to a regimen can only increase the genetic barrier to the regimen.

### Statistical analysis

For classifying therapies as failures versus successes, univariate, multivariate, and regularized multivariate logistic regression was used. For a set of precitors 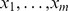, the therapeutic success probability 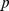 is modeled by the regression
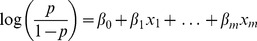
(10)where 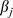 are the regression coefficients. The odds ratio of therapeutic success associated with a one-unit increase in predictor 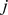 is 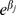. P-values for the predictors are corrected for multiple testing using the Benjamini-Hochberg procedure. For regularization, we applied the elastic net [55], which combines an 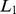 (lasso) penalty encouraging sparse solutions with an 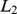 (ridge) penalty that tends to average across correlated features. Classifier performance was evaluated using ROC curves and is reported as the area under the ROC curve (AUC). The data was ten times randomly split into 40% for estimation of the two hyperparameters (one for the degree of each type of regularization) and 60% for model fitting and testing, which was done by 10-fold cross-validation [56].

The R language for statistical computing (http://www.r-project.org/) was used for all analyses, including the R packages icbn, glmnet, and ROCR. An R script for computing the IGB is available at: http://www.cbg.ethz.ch/software/igb. The Stanford HIVDB Sierra web service was used for GSS computation.

## Acknowledgments

We thank the patients who participated in the SHCS; the physicians and study nurses for excellent patient care; the resistance laboratories for high-quality genotypic drug resistance testing; SmartGene, Zug, Switzerland, for technical support; Brigitte Remy, Martin Rickenbach, F. Schoeni-Affolter, and Yannick Vallet from the SHCS Data Center in Lausanne for data management; and Daniéle Perraudin and Mirjam Minichiello for administrative assistance.

The members of the Swiss HIV Cohort Study are: Aubert V, Barth J, Battegay M, Bernasconi E, Böni J, Bucher HC, Burton-Jeangros C, Calmy A, Cavassini M, Egger M, Elzi L, Fehr J, Fellay J, Francioli P, Furrer H (Chairman of the Clinical and Laboratory Committee), Fux CA, Gorgievski M, Günthard H (President of the SHCS), Haerry D (deputy of “Positive Council”), Hasse B, Hirsch HH, Hirschel B, Hösli I, Kahlert C, Kaiser L, Keiser O, Kind C, Klimkait T, Kovari H, Ledergerber B, Martinetti G, Martinez de Tejada B, Metzner K, Müller N, Nadal D, Pantaleo G, Rauch A (Chairman of the Scientific Board), Regenass S, Rickenbach M (Head of Data Center), Rudin C (Chairman of the Mother & Child Substudy), Schmid P, Schultze D, Schöni-Affolter F, Schüpbach J, Speck R, Taffé P, Tarr P, Telenti A, Trkola A, Vernazza P, Weber R, Yerly S.

## Supporting Information

Figure S1 **Treatment change episode (TCE).** Each TCE consists of a failing therapy followed by a salvage therapy. The failing therapy gives rise to a failure, whereas the salvage therapy can be either a success or a failure, depending on whether viral load suppression below 50 cps/ml (400 cps/ml) was achieved during treatment or not (see Methods). Genotypes are measured prior to or at the beginning of the salvage regimen. Examples of successful salvage therapy and failing salvage therapy are given in part (A) and (B) of this figure, respectively.
(EPS)

Figure S2 **Drug usage in the SHCS database.** Drug frequencies among successful (green) and failing (red) regimens for the TCEs of the SHCS database. Successful treatment was defined as a reduction in viral load below 50 cps/ml (A) or 400 cps/ml (B).
(EPS)

Figure S3 **Most abundant drug combinations in the SHCS database.** Frequencies of the 30 most abundant drug combinations in the SHCS database. Successful treatment was defined as a reduction in viral load below 50 cps/ml (A) or 400 cps/ml (B).
(EPS)

Figure S4 **I-CBN model for resistance development to ZDV.** Partially ordered set of RT mutations 41L, 67N, 70R, 74I, 74V, 184V, 210W, 215F, 215Y, 219Q associated with resistance to ZDV (A) and induced genotype lattice (B). Genotypes are colored green if predicted susceptible and red if predicted resistant.
(EPS)

Figure S5 **I-CBN model for resistance development to DDI.** Partially ordered set of RT mutations 41L, 65R, 69Ins, 74VI, 151M, 184VI, 210W, 215FY associated with resistance to DDI (A) and induced genotype lattice (B). Genotypes are colored green if predicted susceptible and red if predicted resistant.
(EPS)

Figure S6 **I-CBN model for resistance development to DDC.** Partially ordered set of RT mutations 41L, 65R, 67N, 75M, 75T, 116Y, 151M, 184V, 210W, 211N associated with resistance to DDC (A) and induced genotype lattice (B). Genotypes are colored green if predicted susceptible and red if predicted resistant.
(EPS)

Figure S7 **I-CBN model for resistance development to D4T.** Partially ordered set of RT mutations 41L, 65R, 67N, 69Ins, 70R, 151M, 184VI, 210W, 215FY, 219QE associated with resistance to D4T (A) and induced genotype lattice (B). Genotypes are colored green if predicted susceptible and red if predicted resistant.
(EPS)

Figure S8 **I-CBN model for resistance development to 3TC.** Partially ordered set of RT mutations 41L, 67N, 70R, 181C, 184V, 190A, 210W, 215F, 215Y, 219Q associated with resistance to 3TC (A) and induced genotype lattice (B). Genotypes are colored green if predicted susceptible and red if predicted resistant.
(EPS)

Figure S9 **I-CBN model for resistance development to ABC.** Partially ordered set of RT mutations 41L, 65R, 69Ins, 74VI, 115F, 151M, 184VI, 210W, 215FY associated with resistance to ABC (A) and induced genotype lattice (B). Genotypes are colored green if predicted susceptible and red if predicted resistant.
(EPS)

Figure S10 **I-CBN model for resistance development to TDF.** Partially ordered set of RT mutations 41L, 65R, 69Ins, 70R, 74VI, 115F, 151M, 184VI, 210W, 215FY associated with resistance to TDF (A) and induced genotype lattice (B). Genotypes are colored green if predicted susceptible and red if predicted resistant.
(EPS)

Figure S11 **I-CBN model for resistance development to FTC.** Partially ordered set of RT mutations 65R, 69Ins, 151M, 184VI associated with resistance to FTC (A) and induced genotype lattice (B). Genotypes are colored green if predicted susceptible and red if predicted resistant.
(EPS)

Figure S12 **I-CBN model for resistance development to EFV.** Partially ordered set of RT mutations 100I, 101EP, 103NS, 106AM, 181CIV, 188LHC, 190ASE, 230L associated with resistance to EFV (A) and induced genotype lattice (B). Genotypes are colored green if predicted susceptible and red if predicted resistant.
(EPS)

Figure S13 **I-CBN model for resistance development to NVP.** Partially ordered set of RT mutations 100I, 101EP, 103NS, 106AM, 181CIV, 188LHC, 190ASE, 230L associated with resistance to NVP (A) and induced genotype lattice (B). Genotypes are colored green if predicted susceptible and red if predicted resistant.
(EPS)

Figure S14 **I-CBN model for resistance development to RTV.** Partially ordered set of PR mutations 24I, 30N, 32I, 46I, 46L, 54V, 73S, 82A, 84V, 90M associated with resistance to RTV (A) and induced genotype lattice (B). Genotypes are colored green if predicted susceptible and red if predicted resistant.
(EPS)

Figure S15 **I-CBN model for resistance development to SQV.** Partially ordered set of PR mutations 48VM, 54VTALM, 82AT, 84V, 88S, 90M associated with resistance to SQV (A) and induced genotype lattice (B). Genotypes are colored green if predicted susceptible and red if predicted resistant.
(EPS)

Figure S16 **I-CBN model for resistance development to IDV.** Partially ordered set of PR mutations 32I, 46IL, 47V, 54VTALM, 76V, 82AFTS, 84V, 88S, 90M associated with resistance to IDV (A) and induced genotype lattice (B). Genotypes are colored green if predicted susceptible and red if predicted resistant.
(EPS)

Figure S17 **I-CBN model for resistance development to NFV.** Partially ordered set of PR mutations 30N, 46IL, 47V, 48VM, 54VTALM, 82AFTS, 84V, 88DS, 90M associated with resistance to NFV (A) and induced genotype lattice (B). Genotypes are colored green if predicted susceptible and red if predicted resistant.
(EPS)

Figure S18 **I-CBN model for resistance development to LPV.** Partially ordered set of PR mutations 32I, 46IL, 47VA, 48VM, 50V, 54VTALM, 76V, 82AFTS, 84V, 90M associated with resistance to LPV (A) and induced genotype lattice (B). Genotypes are colored green if predicted susceptible and red if predicted resistant.
(EPS)

Figure S19 **I-CBN model for resistance development to APV.** Partially ordered set of PR mutations 24I, 32I, 46I, 46L, 48V, 53L, 54V, 82A, 84V, 90M associated with resistance to APV (A) and induced genotype lattice (B). Genotypes are colored green if predicted susceptible and red if predicted resistant.
(EPS)

Figure S20 **I-CBN model for resistance development to ATV.** Partially ordered set of PR mutations 10I, 32I, 33F, 46I, 48V, 54V, 71V, 82A, 84V, 90M associated with resistance to ATV (A) and induced genotype lattice (B). Genotypes are colored green if predicted susceptible and red if predicted resistant.
(EPS)

Figure S21 **I-CBN model for resistance development to TPV.** Partially ordered set of PR mutations 32I, 46IL, 47VA, 54VAM, 82TL, 84V associated with resistance to TPV (A) and induced genotype lattice (B). Genotypes are colored green if predicted susceptible and red if predicted resistant.
(EPS)

Figure S22
**Univariate analysis of predictors of response to antiretroviral combination therapy in the SHCS database.** Associations have been tested using logistic regression models and odds ratios of therapeutic success, defined as viral load reduction below 50cps/ml (A) and 400cps/ml (B), are reported together with their 95% confidence intervals on a logarithmic scale. Benjamini-Hochberg-corrected p-values are represented as black (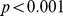) and grey (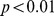) symbols. Only predictors with a p-value smaller than 0.01 are included.(EPS)

Table S1 **Complete list of all variables analyzed with respect to treatment outcome.** Groups NRTI, NNRTI, and PI consist of binary variables, one for each drug, indicating the presence of the respective drug in the regimen. For PIs, boosted (given together with low-dose RTV) and unboosted formulations are distinguished, except for LPV which is always applied boosted. The variable RTV refers to the use of ritonavir as the only PI in the regimen. Demographic and clinical variables include age and gender of the patient, whether he or she had AIDS, the maximum viral load and the minimum CD4 T cell count measured anytime before treatment onset, transmission group (BLOOD, HET, IDU, MSM, or OTHER), and adherence. Patient adherence was assessed in questionnaires and measured as the percentage of missed dosages [50], [51] for 1183 (45%) of the patients, and then dichotomized. For the multivariate analysis only, unobserved values of patient adherence were imputed by a logistic regression model (one for each dataset) from all remaining variables except the response (treatment outcome). For each drug, the individualized genetic barrier (IGB) is the probability of the virus not escaping from the selective pressure of the drug. The IGB to regimen is defined as the sum of the drug-specific IGBs over all drugs in the regimen. Mutations in the PR and RT of HIV-1 are denoted by the sequence position followed by the amino acid. Each variable is binary indicating the presence of the respective amino acid at the respective position in the protein. Only mutations that occurred in at least 5% of the samples are considered.
(PDF)

Table S2
**Construction of I-CBN models.** For each drug, is reported the number 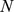 of genotype-phenotype pairs the model has been learned from, the correlation coefficient 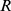 between predicted and true drug resistance phenotypes, the list of selected mutations, and the cutoff value 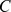 defining resistant versus susceptible viruses. The correlation coefficient has been estimated from an independent test set consisting of 20% of the data that was not used for training. For ZDV, DDI, D4T, 3TC, ABC, TDF, FTC, EFV, NVP, SQV, IDV, NFV, LPV, and TPV, the corresponding drug resistance-associated mutations reported on the Stanford HIV Drug Resistance Database website were used, while for DDC, RTV, APV, and ATV, we selected ten mutations using L1-penalized linear regression (lasso).(PDF)

Table S3 **Different categories of drug combinations in SHCS databse.** The first category includes drug combinations currently recommended as first-line or alternative regimens according to the JAMA recommendations [42]. Category 2 includes regimens that were recommended as first-line or second-line regimens in the past, regimens that are still in use in developing countries or are used sometimes if drug resistant virus is present at baseline, or salvage regimens. Category 3 includes older regimens that are not in use anymore as first-line regimens but were before, regimens that are not corresponding to guidelines, including those that are sometimes used in special circumstances, such as unusual tolerability, etc. To evaluate the prediction performance (sensitivity and specificity) of each category, leave-one-out cross-validation experiments were performed.
(PDF)

Table S4 **PI usage and boosting fraction.** Reported is the total number of regimens in the SHCS database that include the respective PI, and in parenthesis, the percentage that the PI is boosted, i.e., given together with low-dose ritonavir (RTV).
(PDF)

Table S5
**Comparative performance in predicting treatment outcome, defined as a reduction of viral load below 50cps/ml, for different elastic net regularized logistic regression models.** Comparative performance in predicting treatment outcome, defined as a reduction of viral load below 50cps/ml, for different elastic net regularized logistic regression models. In columns 3–8, the 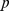-value of a two-sided Wilcoxon rank sum test for differences in the area under the ROC curve (AUC; column 2) is reported. Prediction models (column 1) are encoded by the sets of predictors used, where C refers to the demographic and clinical variables, D refers to drugs, and M to mutations. For example, the model IGB+CDM includes as predictors IGB to regimen, clinical and demographic predictors, applied drugs, and mutations.(PDF)

Table S6
**Comparative performance in predicting treatment outcome, defined as a reduction of viral load below 400cps/ml, for different elastic net regularized logistic regression models.** Comparative performance in predicting treatment outcome, defined as a reduction of viral load below 400cps/ml, for different elastic net regularized logistic regression models. In columns 3–8, the 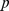-value of a two-sided Wilcoxon rank sum test for differences in the area under the ROC curve (AUC; column 2) is reported. Prediction models (column 1) are encoded by the sets of predictors used, where C refers to the demographic and clinical variables, D refers to drugs, and M to mutations. For example, the model IGB+CDM includes as predictors IGB to regimen, clinical and demographic predictors, applied drugs, and mutations.(PDF)
