# Supplementary material for: The Individualized Genetic Barrier Predicts Treatment Response in a Large Cohort of HIV-1 Infected Patients
Source: PLoS Comput Biol. 2013 Aug 29;9(8):e1003203. doi: 10.1371/journal.pcbi.1003203 (PMC3757085; doi:10.1371/journal.pcbi.1003203)
Supplement: Table S1 — Complete list of all variables analyzed with respect to treatment outcome. Groups NRTI, NNRTI, and PI consist of binary variables, one for each drug, indicating the presence of the respective drug in the regimen. For PIs, boosted (given together with low-dose RTV) and unboosted formulations are distinguished, except for LPV which is always applied boosted. The variable RTV refers to the use of ritonavir as the only PI in the regimen. Demographic and clinical variables include age and gender of the patient, whether he or she had AIDS, the maximum viral load and the minimum CD4 T cell count measured anytime before treatment onset, transmission group (BLOOD, HET, IDU, MSM, or OTHER), and adherence. Patient adherence was assessed in questionnaires and measured as the percentage of missed dosages [50], [51] for 1183 (45%) of the patients, and then dichotomized. For the multivariate analysis only, unobserved values of patient adherence were imputed by a logistic regression model (one for each dataset) from all remaining variables except the response (treatment outcome). For each drug, the individualized genetic barrier (IGB) is the probability of the virus not escaping from the selective pressure of the drug. The IGB to regimen is defined as the sum of the drug-specific IGBs over all drugs in the regimen. Mutations in the PR and RT of HIV-1 are denoted by the sequence position followed by the amino acid. Each variable is binary indicating the presence of the respective amino acid at the respective position in the protein. Only mutations that occurred in at least 5% of the samples are considered. (PDF) [file pcbi.1003203.s023.pdf]

**Group**	**Variables**
NRTI	ZDV, DDI, DDC, D4T, 3TC, ABC, TDF, FTC
NNRTI	EFV, NVP
PI	RTV, SQV, SQV/r, IDV, IDV/r, NFV, NFV/r, LPV/r, APV, APV/r, ATV, ATV/r, TPV, TPV/r
Demographic Variables	Age, Gender, Transmission group
Clinical Variables	AIDS, Maximum viral load, Minimum CD4 T cell count, Adherence to treatment
IGB	IGB to ZDV, IGB to DDI, IGB to DDC, IGB to D4T, IGB to 3TC, IGB to ABC, IGB to TDF, IGB to FTC, IGB to EFV, IGB to NVP, IGB to RTV, IGB to SQV, IGB to IDV, IGB to NFV, IGB to LPV, IGB to APV, IGB to ATV, IGB to TPV, IGB to regimen
PR	10F, 10I, 10V, 13V, 14R, 15V, 16E, 19I, 20I, 20R, 20T, 30N, 33F, 35D, 36I, 37D, 37H, 41K, 46I, 46L, 54V, 57K, 60E, 62V, 63P, 63S, 63T, 64V, 69K, 70R, 71T, 71V, 72T, 72V, 73S, 74S, 77I, 82A, 84V, 88D, 89M, 90M, 93L
RT	6D, 20R, 35I, 35M, 35T, 36A, 39A, 39E, 40D, 41L, 43E, 44D, 49R, 60I, 62V, 67N, 70R, 74V, 83K, 90I, 98G, 98S, 103N, 108I, 118I, 121H, 122K, 123E, 123N, 123S, 135T, 135V, 142V, 162A, 162C, 165I, 166R, 169D, 173A, 173T, 174K, 177E, 178L, 178M, 179I, 181C, 184V, 190A, 196E, 200A, 200I, 202V, 203K, 207A, 207E, 208Y, 210W, 211K, 211S, 215F, 215Y, 219E, 219Q, 245E, 245Q, 248D, 250E, 281R, 283I, 286A, 291D, 292I, 293V, 294T, 297A, 297K, 297R, 311R, 317A, 322T, 324E, 326V, 329L, 329V, 334L, 335D, 356K, 359S, 371V, 390R
