# Supplementary material for: The Individualized Genetic Barrier Predicts Treatment Response in a Large Cohort of HIV-1 Infected Patients
Source: PLoS Comput Biol. 2013 Aug 29;9(8):e1003203. doi: 10.1371/journal.pcbi.1003203 (PMC3757085; doi:10.1371/journal.pcbi.1003203)
Supplement: Table S2 — Construction of I-CBN models. For each drug, is reported the number of genotype-phenotype pairs the model has been learned from, the correlation coefficient between predicted and true drug resistance phenotypes, the list of selected mutations, and the cutoff value defining resistant versus susceptible viruses. The correlation coefficient has been estimated from an independent test set consisting of 20% of the data that was not used for training. For ZDV, DDI, D4T, 3TC, ABC, TDF, FTC, EFV, NVP, SQV, IDV, NFV, LPV, and TPV, the corresponding drug resistance-associated mutations reported on the Stanford HIV Drug Resistance Database website were used, while for DDC, RTV, APV, and ATV, we selected ten mutations using L1-penalized linear regression (lasso). (PDF) [file pcbi.1003203.s024.pdf]

Drug	Class	N	R	Selected mutations	C
ZDV	NRTI	1392	0.604	41L, 65R, 67N, 69Ins, 70R, 74VI, 151M, 184VI, 210W, 215FY, 219QE	30.0
DDI	NRTI	1520	0.686	41L, 65R, 69Ins, 74VI, 151M, 184VI, 210W, 215FY	2.4
DDC	NRTI	1135	0.594	41L, 65R, 67N, 75M, 75T, 116Y, 151M, 184V, 210W, 211N	2.2
D4T	NRTI	1460	0.641	41L, 65R, 67N, 69Ins, 70R, 151M, 184VI, 210W, 215FY, 219QE	2.0
3TC	NRTI	893	0.853	41L, 67N, 70R, 181C, 184V, 190A, 210W, 215F, 215Y, 219Q	15.4
ABC	NRTI	1348	0.652	41L, 65R, 69Ins, 74VI, 115F, 151M, 184VI, 210W, 215FY	3.4
TDF	NRTI	942	0.58	41L, 65R, 69Ins, 70R, 74VI, 115F, 151M, 184VI, 210W, 215FY	2.1
FTC	NRTI	290	0.669	65R, 69Ins, 151M, 184VI	17.0
EFV	NNRTI	1212	0.82	100I, 101EP, 103NS, 106AM, 181CIV, 188LHC, 190ASE, 230L	7.0
NVP	NNRTI	1134	0.824	100I, 101EP, 103NS, 106AM, 181CIV, 188LHC, 190ASE, 230L	9.0
RTV	PI	1821	0.812	24I, 30N, 32I, 46I, 46L, 54V, 73S, 82A, 84V, 90M	2.6
SQV	PI	2124	0.701	48VM, 54VTALM, 82AT, 84V, 88S, 90M	4.5
IDV	PI	2170	0.785	32I, 46IL, 47V, 54VTALM, 76V, 82AFTS, 84V, 88S, 90M	4.6
NFV	PI	2135	0.782	30N, 46IL, 47V, 48VM, 54VTALM, 82AFTS, 84V, 88DS, 90M	5.8
LPV	PI	1875	0.869	32I, 46IL, 47VA, 48VM, 50V, 54VTALM, 76V, 82AFTS, 84V, 90M	10.0
APV	PI	1494	0.717	24I, 32I, 46I, 46L, 48V, 53L, 54V, 82A, 84V, 90M	12.0
ATV	PI	1473	0.661	10I, 32I, 33F, 46I, 48V, 54V, 71V, 82A, 84V, 90M	4.2
TPV	PI	1458	0.584	32I, 46IL, 47VA, 54VAM, 82TL, 84V	2.5
