# Supplementary material for: The Individualized Genetic Barrier Predicts Treatment Response in a Large Cohort of HIV-1 Infected Patients
Source: PLoS Comput Biol. 2013 Aug 29;9(8):e1003203. doi: 10.1371/journal.pcbi.1003203 (PMC3757085; doi:10.1371/journal.pcbi.1003203)
Supplement: Table S3 — Different categories of drug combinations in SHCS databse. The first category includes drug combinations currently recommended as first-line or alternative regimens according to the JAMA recommendations [42]. Category 2 includes regimens that were recommended as first-line or second-line regimens in the past, regimens that are still in use in developing countries or are used sometimes if drug resistant virus is present at baseline, or salvage regimens. Category 3 includes older regimens that are not in use anymore as first-line regimens but were before, regimens that are not corresponding to guidelines, including those that are sometimes used in special circumstances, such as unusual tolerability, etc. To evaluate the prediction performance (sensitivity and specificity) of each category, leave-one-out cross-validation experiments were performed. (PDF) [file pcbi.1003203.s025.pdf]

Drug combinations		50 cps/ml				400 cps/ml		
			sensitvity	specificity			sensitvity	specificity
Total	2,185	(100%)	0.54	0.91	2,631	(100%)	0.52	0.89
Category 1	206	(9%)	0.65	0.62	218	(8%)	0.69	0.66
Category 2	385	(18%)	0.62	0.80	438	(17%)	0.62	0.79
Category 3	1,594	(73%)	0.47	0.95	1,975	(75%)	0.44	0.93
Unique	426				465			
