# Supplementary material for: The Individualized Genetic Barrier Predicts Treatment Response in a Large Cohort of HIV-1 Infected Patients
Source: PLoS Comput Biol. 2013 Aug 29;9(8):e1003203. doi: 10.1371/journal.pcbi.1003203 (PMC3757085; doi:10.1371/journal.pcbi.1003203)
Supplement: Table S4 — PI usage and boosting fraction. Reported is the total number of regimens in the SHCS database that include the respective PI, and in parenthesis, the percentage that the PI is boosted, i.e., given together with low-dose ritonavir (RTV). (PDF) [file pcbi.1003203.s026.pdf]

**PI (boosted)**	**50 cps/ml**		**400 cps/ml**	
SQV	205	(58%)	262	(59%)
IDV	168	(27%)	239	(26%)
NFV	360	(0%)	464	(0%)
APV	108	(41%)	130	(41%)
LPV	467	(100%)	545	(100%)
ATV	238	(82%)	250	(81%)
TPV	21	(100%)	22	(100%)
DRV	27	(96%)	27	(96%)
