# Supplementary material for: The Individualized Genetic Barrier Predicts Treatment Response in a Large Cohort of HIV-1 Infected Patients
Source: PLoS Comput Biol. 2013 Aug 29;9(8):e1003203. doi: 10.1371/journal.pcbi.1003203 (PMC3757085; doi:10.1371/journal.pcbi.1003203)
Supplement: Table S5 — Comparative performance in predicting treatment outcome, defined as a reduction of viral load below 50cps/ml, for different elastic net regularized logistic regression models. Comparative performance in predicting treatment outcome, defined as a reduction of viral load below 50cps/ml, for different elastic net regularized logistic regression models. In columns 3–8, the -value of a two-sided Wilcoxon rank sum test for differences in the area under the ROC curve (AUC; column 2) is reported. Prediction models (column 1) are encoded by the sets of predictors used, where C refers to the demographic and clinical variables, D refers to drugs, and M to mutations. For example, the model IGB+CDM includes as predictors IGB to regimen, clinical and demographic predictors, applied drugs, and mutations. (PDF) [file pcbi.1003203.s027.pdf]

	**AUC**	**IGB**	**GSS**	**IGB+C**	**GSS+C**	**IGB+CDM**	**GSS+CDM**
**IGB**	0.576						
**GSS**	0.621	0.0058					
**IGB+C**	0.618	0.0049	0.55				
**GSS+C**	0.662	2e-07	0.044	0.0012			
**IGB+CDM**	0.851	7e-35	5e-32	1e-33	1e-30		
**GSS+CDM**	0.858	4e-35	6e-33	4e-34	9e-32	0.13	
**GSS+IGB+CDM**	0.861	4e-35	3e-33	4e-34	3e-32	0.021	0.34
