# Supplementary material for: The Individualized Genetic Barrier Predicts Treatment Response in a Large Cohort of HIV-1 Infected Patients
Source: PLoS Comput Biol. 2013 Aug 29;9(8):e1003203. doi: 10.1371/journal.pcbi.1003203 (PMC3757085; doi:10.1371/journal.pcbi.1003203)
Supplement: Table S6 — Comparative performance in predicting treatment outcome, defined as a reduction of viral load below 400cps/ml, for different elastic net regularized logistic regression models. Comparative performance in predicting treatment outcome, defined as a reduction of viral load below 400cps/ml, for different elastic net regularized logistic regression models. In columns 3–8, the -value of a two-sided Wilcoxon rank sum test for differences in the area under the ROC curve (AUC; column 2) is reported. Prediction models (column 1) are encoded by the sets of predictors used, where C refers to the demographic and clinical variables, D refers to drugs, and M to mutations. For example, the model IGB+CDM includes as predictors IGB to regimen, clinical and demographic predictors, applied drugs, and mutations. (PDF) [file pcbi.1003203.s028.pdf]

	**AUC**	**IGB**	**GSS**	**IGB+C**	**GSS+C**	**IGB+CDM**	**GSS+CDM**
**IGB**	0.648						
**GSS**	0.638	0.38					
**IGB+C**	0.664	0.1	0.97				
**GSS+C**	0.741	1e-16	1e-07	1e-12			
**IGB+CDM**	0.829	2e-33	2e-31	1e-33	4e-28		
**GSS+CDM**	0.816	8e-33	1e-29	1e-32	1e-24	0.017	
**GSS+IGB+CDM**	0.835	7e-34	1e-32	6e-34	8e-30	0.22	2e-04
